# Breath Tests in Respiratory and Critical Care Medicine: From Research to Practice in Current Perspectives

**DOI:** 10.1155/2013/702896

**Published:** 2013-09-18

**Authors:** Attapon Cheepsattayakorn, Ruangrong Cheepsattayakorn

**Affiliations:** ^1^10th Zonal Tuberculosis and Chest Disease Center, Chiang Mai, 10th Office of Disease Prevention and Control, Department of Disease Control, Ministry of Public Health, Chiang Mai 50100, Thailand; ^2^Department of Pathology, Faculty of Medicine, Chiang Mai University, Chiang Mai 50200, Thailand

## Abstract

Today, exhaled nitric oxide has been studied the most, and most researches have now focusd on asthma. More than a thousand different volatile organic compounds have been observed in low concentrations in normal human breath. Alkanes and methylalkanes, the majority of breath volatile organic compounds, have been increasingly used by physicians as a novel method to diagnose many diseases without discomforts of invasive procedures. None of the individual exhaled volatile organic compound alone is specific for disease. Exhaled breath analysis techniques may be available to diagnose and monitor the diseases in home setting when their sensitivity and specificity are improved in the future.

## 1. Introduction

 Acetaldehyde breath test has been used to measure the production of this compound after ingestion of ethanol, but its interpretation is affected by many factors: ALDH2 polymorphism, alcoholic drinking habits, and smoking which are correlated with higher breath and blood levels [1]. The psychological expectancies of alcoholic drinking are more positive and less negative for ALDH2*1/*2 genotype individuals with alcoholism, although the ALDH2*2 allele has been correlated with negative physiological responses in normal subjects [2]. Some authors have concluded that low levels of exhaled acetaldehyde cannot be used to measure blood levels [1]. Various pulmonary diseases involve oxidative stress and chronic inflammation [3]. These are not yet measured directly in routine clinical practice because of the difficulties in monitoring inflammation [3]. It is unsuitable for repeated use of fiberoptic bronchial biopsies as the “gold standard” and histamine or methacholine challenge for monitoring inflammation in patients with severe asthma and in children [3]. The interpretation of histamine or methacholine challenge in asthma measurement of airway inflammation may be confounded by the use of bronchodilator therapy [3]. This has led to the use of induced sputum to detect inflammation [4]. However, this technique is invasive and cannot repeat measurements in less than 24 hours [4]. The need to monitor pulmonary inflammation has led to the exploration of exhaled markers and condensates [5]. Breath test is presently a research procedure, but it may have an important place in the diagnosis and management of pulmonary diseases in the future [5]. 

 More than a thousand different volatile organic compounds (VOCs) have been observed in low concentrations in normal human breath [6]. Despite this unflattering association with inebriation and arrest, breath testing is blossoming into an exciting area of medical technology [7]. Breath tests have attracted a great amount of clinical and scientific interests during the last decade [8]. Physicians have begun using these tests to diagnose an increasing wide variety of diseases without the hazards or discomforts of invasive procedures [7]. Furthermore, breath tests are providing important new insights into the understanding of basic biochemical functions of the body [7]. However, its sensitivity, variability, and reproducibility need to be addressed before it can be recommended as an outcome monitoring. Many of the new assays are now in development for predicting disease progression and response to present and novel therapies including indicating the disease instability. 

## 2. Exhaled Markers in Pulmonary Tuberculosis

A study for breath biomarkers of tuberculosis (TB) was conducted among 23 and 19 patients with positive and negative sputum cultures for *Mycobacterium tuberculosis* (*M tbc*), respectively, [9]. One hundred and thirty different VOCs which are mainly bloodborne generated were consistently detected in *M tbc* cultures in vitro, predominantly derivatives of benzene, naphthalene, and alkanes [9]. Naphthalene,1-methyl- and cyclohexane, 1,4-dimethyl- were observed both in vitro and breath [9]. Major VOCs which significantly increased in all hospitalized patients compared to the healthy controls were 1,3-isobenzofurandione, pentane, 2,3-dimethyl-, acetaldehyde, benzenemethanol,_alpha_,_alpha_-dimethyl-,cyclohexane, 1,1′-biphenyl, 2,2′-diethyl-, and 1h-indene, 2,3-dihydro-1,1,3-trimethyl-3-phenyl- [9]. Pattern recognition analysis and fuzzy logic analysis of breath VOCs independently distinguished healthy controls from hospitalized patients with 100% sensitivity and 100% specificity [9]. Pattern recognition analysis identified patients with positive sputum cultures with 82.6% sensitivity and 100% specificity [9]. Accordingly, the *M tbc* urease enzyme encoded by *ureA* (*Rv1848*), *ureB* (*Rv1849*), and *ureC* (*Rv1850*) hydrolyzes urea into carbon dioxide and ammonia [10]. An attractive point of urease-based diagnostics is that humans lack urease enzymes [11]. Since 2009, a group of investigators started studying the benefits of rabbit urease breath test for TB diagnosis (UBT-TB) and treatment monitoring [11]. The specificity of UBT-TB may be increased by introducing intravenous or inhaled ^13^C-urea tracer, thus preventing gastric *Helicobacter* species contaminations [11]. Anti-TB chemotherapy in the first two months and oral nonabsorbable urease inhibitors such as bismuth subsalicylate or proton pump inhibitors have also increased the specificity of UBT-TB for TB treatment monitoring by suppression of gastrointestinal *Helicobacter pylori* [12]. Thus, UBT-TB can be applied for diagnosis and treatment monitoring of human pulmonary TB [12, 13]. A recent study on point-of-care (POC) breath test demonstrated that POC-breath test can detect VOCs which are the metabolic products, particularly derivatives of benzene, alkanes, and naphthalene of *M tbc* in patients with active pulmonary TB with 80% accuracy, 71.2% sensitivity, and 72% specificity [14]. In age-matched subgroup, the accuracy increased to 84%, and in a population with 5% prevalence, the POC-breath test detected active pulmonary TB with 13% positive predictive value and 98% negative predictive value [14]. Two previous studies revealed that gas chromatography-mass spectrometry (GC-MS) breath test can diagnose TB with 77% and 85% accuracies, 62% and 84% sensitivities and 84%, and 64.7% specificities, respectively [15, 16], whereas a recent study showed very low concentrations of specific volatiles detectable by the same diagnostic tool for *M tbc*, *Aspergillus fumigatus*, and *Pseudomonas aeruginosa* [17].

 Other several species of Halic produce VOC metabolites that act as chemical “fingerprints”: *M. avium*, *M. gordonae*, *M. gastri*, *M. kansasii*, *M. szulgai*, and *M. flavescens* can be identified by their distinctive patterns of volatile metabolites, including C14–C26 fatty acids and their methylated and hydroxylated derivatives [18–20]. A study of clinical isolates of Halic with gas-liquid chromatography alone demonstrated that all strains of *M. gordonae* and *M. kansasii* were identified to species level [18].* M tbc* was definitely identified in 85% of the cases [18]. When it could not be definitely identified, the only alternatives were *M. bovis* and *M. xenopi*, both of which are rare causes of infection [18]. A recent study demonstrated that pulsed discharge helium ionization detector can detect o-phenyl anisole, methyl phenylacetate, methyl p-anisate and methyl nicotinate which are potential volatile biomarkers for species of Halic [21]. 

A study of exhaled hydrogen peroxide (H_2_O_2_) level in patients with pulmonary TB revealed significantly decreased after 2 months of successful antituberculous treatment and its level did not correlate with circulating interleukin (IL)-18 in TB patients before or after treatment compared with control groups [22]. Nitric oxide (NO) is mainly generated in the bronchial system [5]. Patients with active TB demonstrated elevation of exhaled NO with NOS2 expression in alveolar macrophages and reduced with antituberculous treatment [23, 24]. Nevertheless, some investigators reported that exhaled NO measurement had limited value in the direct diagnosis of pulmonary TB, although it revealed 84% sensitivity and 67% specificity, but it may be worth developing as a cost-effective replacement of chest radiography in screening algorithms of pulmonary TB, where chest radiography is not available [25].

## 3. Exhaled Markers in Pulmonary Cancer

 Alkanes and monomethylated alkanes are oxidative stress products that are excreted in the breath, the catabolism which may be accelerated by polymorphic cytochrome p450-mixed oxidase enzymes (CYP) that are induced in patients with pulmonary cancer and result in measurable changes in the composition of the breath [26]. In 2003, a study reported a breath test for C4 to C20 alkanes and monomethylated alkanes that provided a rational set of markers which identified pulmonary cancer in a group of patients with histologically proven disease [26]. Patients with pulmonary cancer demonstrated significantly high level of exhaled NO with the intensity of NOS2 expression in alveolar macrophage [27].

 In 2007, a study concluded that a two-minute breath test can predict pulmonary cancer with accuracy comparable to screening chest computed tomography [28]. The accuracy of the test was not affected by TNM (tumor, node, and metastasis) stage of disease or tobacco smoking [28]. 

 Alterations in breath VOCs in pulmonary cancer were consistent with a nonlinear pathophysiologic process, such as an off-on switch controlling high-risk CYP activity [28]. VOCs breath test with weighted digital analysis predicted pulmonary cancer with accuracy the same as chest computed tomography [29]. Its accuracy was not affected by tobacco smoking and TMN stage of disease [29]. In 2005, a study concluded that none of the exhaled VOCs alone was specific for pulmonary cancer; a combination of 13 VOCs does allow the classification of cases into groups [30]. A study using an electronic nose showed additionally different VOCs within different types of pulmonary cancer cells, especially 4 special VOCs that were demonstrated to exist in all culture media of cancer cells [31]. 

Exhaled VOC analysis may therefore be useful in improving the specificity and sensitivity of conventional diagnostic approach to pulmonary cancer [30]. A study showed that 67 VOCs were common to the breath samples of 62 (57.4%) of pulmonary cancer patients [32]. The mean posttest probability of pulmonary cancer for combination of 22 breath VOCs, predominantly alkanes, alkane derivatives, and benzene derivatives in breath samples of patients with an abnormal chest roentgenogram was significantly higher in patients with pulmonary cancer than in those without pulmonary cancer (all stages *P* < 0.0003) [32]. In patients with stage I pulmonary cancer, a posttest probability of 0.46 had 100% sensitivity and 81.3% specificity; a posttest probability over 0.90 had 66.7% sensitivity and 100% specificity [32]. Exhaled breath analysis can discriminate benign from malignant pulmonary nodules; furthermore, it can discriminate squamous-cell carcinoma and adenocarcinoma and early versus advanced disease stages [33]. A recent study demonstrated that exhaled breath tests with solid phase microextraction (SPME)-gas chromatography (GC)-Mass Spectroscopy (MS) can detect VOCs biomarkers for pulmonary cancer with 96.47% sensitivity and 97.47% specificity [34]. The diagnosis was correctly predicted by combination of age, tobacco smoking, and sex in 65.7% of cases, compared with 81.5% by the breath VOCs [32]. Cross-validation correctly predicted the diagnosis in 71.1% of patients with pulmonary cancer and 66.7% of those without pulmonary cancer [32]. In 2007, a study showed that sampling of exhaled NO can be used as a large screening test for pulmonary cancer [35] and another study demonstrated higher exhaled breath condensate (EBC) levels of IL-2, tumor necrosis factor (TNF)-alpha, and leptin in patients from stages 1 to 3 of nonsmall cell pulmonary cancer [36]. Exhaled matrix metalloproteinase-9 (MMP-9) in patients with progressive nonsmall cell pulmonary cancer measured in EBC was indicated to be significantly higher as well as in pleural effusion and whole blood and demonstrated positive correlation between MMP-9 levels in EBC, cigarette smoking status, and stage of pulmonary cancer [37]. A recent study of microsatellite alterations (MAs) of 3p in the EBC deoxyribonucleic acid (DNA) of patients with nonsmall cell pulmonary cancer revealed that MAs in EBC DNA of cancer patients were significantly more common than in normal subjects and MA profile of EBC DNA corresponded to that from cancer tissue of each patient [38]. Isoprene and acetone were shown to increase in the exhaled breath measured by SPME-GC-MS breath test after cytostatic chemotherapy indicating the usefulness of this technique in monitoring of pulmonary cancer-chemotherapy efficacy [39]. Some recent studies revealed reduction of mutated *KRAS* oncogene in EBC [40] and some VOCs in exhaled breath [41] after surgical resection of pulmonary tumor, particularly nonsmall cell type. Unfortunately, a recent study demonstrated that serial FE_NO_ measurements during radiotherapy in patients with pulmonary cancer had a low ability to detect symptomatic radiation pneumonitis [42]. 

## 4. Exhaled Markers in Respiratory Infections

 In 2005, a study reported that notably hydrogen cyanide (HCN) gas concentration was significantly higher above *Pseudomonas aeruginosa* (*P. aeruginosa*)-positive specimens than above other bacterial growths (*P* < 0.01), and levels of HCN greater than 100 part per billion were a sensitive (68%) and highly specific (100%) VOC of *P. aeruginosa *[43]. Increased exhaled CO levels with significantly decreasing after treatment were shown in patients with bacterial lower respiratory tract infection [44]. Elevated exhaled CO levels were also found in children [45] and adults [46] with upper respiratory tract viral infections. Decreased exhaled and nasal NO levels were seen in human immunodeficiency virus infected patients and more decreased in progressive cases [47]. Low exhaled and nasal NO levels were demonstrated in primary ciliary dyskinesia (PCD) or cystic fibrosis (CF) patients with recurrent bacterial or viral pulmonary infections or recurrent pulmonary parasitic infestations and patients with active Wegener's granulomatosis infected with *Staphylococcus aureus* [48]. The increasing of exhaled NO levels was observed in patients with chronic bronchitis [49] and bacterial upper [50] and lower [49] respiratory tract inflammations. In adults, NO is produced by the paranasal sinuses and its exhaled concentrations elevate in normal subjects after digested, intravenous, or inhaled L-arginine [51]. In normal subjects, nasal air contains only NO, whereas orally exhaled air contains both NO and CO [52]. A study demonstrated that 2-pentylfuran may be an exhaled biomarker for detection of pulmonary fungal and *Streptococcus pneumoniae* infections [53]. Higher concentrations of volatile fatty acids in the bronchoalveolar lavage (BAL) were observed in the patients with pneumonia infected with fermenting anaerobic bacteria which are neither found by microscopy nor cultivation [54].

## 5. Exhaled Markers in Some Pulmonary Diseases

 Exhaled NO levels are increased in atopic asthma, normal in chronic obstructive pulmonary diseases (COPD) and asbestosis [55, 56], and reduced in CF and in PCD [57]. Elevated levels of EBC 8-isoprostane in both asbestosis and silicosis of leukotriene (LT) B4 in asbestosis and of LT D4 in silicosis were also observed [56, 58]. Smoking is a factor which decreases exhaled NO levels [59] but a study showed statistically significant small and transient elevation in fractional exhaled NO (FE_NO_) levels [60]. A recent study demonstrated that there was no statistically significant difference in FE_NO_ concentrations between asthmatic and nonasthmatic patients with nasal polyposis [61]. FE_NO_ was reduced by effects of both soluble and suspension formulations of nebulized budesonide in asthmatic children [62]. Patients with stable COPD are the same as the healthy smoking subjects [59]. Exhaled carbon monoxide (CO) is increased in asthma, CF, and COPD [57]. A study demonstrated no significant elevation of exhaled CO levels in patients with either allergic rhinitis or CF, or steroid-treated or steroid-naïve asthma, as compared with controls [63]. In inhaled-corticosteroid- (ICS-) treated asthma patients, normal exhaled CO level was reported [64]. Raising and reduction of exhaled pentane were shown during acute asthma exacerbations and recovery, respectively, [65]. When compared with control subjects and steroid-treated patients, higher exhaled ethane levels were demonstrated in mild steroid-naïve asthma patients [66]. In 2007, a study revealed that an electronic nose, a chemical sensor array, can discriminate exhaled breath of patients with asthma from healthy controls but is less accurate in distinguishing asthma severities [67].

 Reduced EBC ammonium ions were observed in patients with acute asthma [68]. Exhaled prostaglandin E_2_ and EBC prostaglandin F_2_-alpha are markedly increased in COPD but not in asthma [69, 70]. In contrast, LT E4 is increased in asthma but is not detectable in COPD patients and in normal subjects [71] as well as EBC thromboxane (Tx) B_2_ (Montuschi et al., unpublished observation). EBC concentrations of prostaglandin D_-_methoxime are the same in normal subjects and COPD patients [69], whereas no Tx B_2_-Ll is detectable in COPD patients [68] but is measurable in about 50% of asthmatic patients [70]. Some studies showed detectable levels of the LT B4, C4, D4, E4, and F4 in EBC of normal and asthmatic subjects [72, 73], especially EBC C4, D4, and E4 in patients with moderate and severe asthma [73] with close correlation with disease severity [74], but no correlation with pulmonary function tests [74]. In patients with moderate asthma with steroid withdrawal lead to elevation of EBC B4, C4, D4, and E4 levels and worsening of asthma [75]. Elevation of B4 levels was demonstrated in EBC of patients with moderate or severe asthma [73], COPD exacerbations [76], and stable COPD [69] and was shown in the sputum of patients with bacterial exacerbations of COPD [74] and bronchiectasis [77] as well. EBC LT B4 may be involved in asthma exacerbations and may contribute towards neutrophils recruitment (Montuschi et al., unpublished observations). Unchanged EBC N (epsilon) (carboxymethyl)lysine levels in nonsmoking asthmatics and significant elevation in smoking asthmatics were observed [78]. Higher EBC Th2-specific macrophage-derived chemokine and eotaxin, an eosinophil chemoattractant, levels were demonstrated in ICS-treated asthmatics compared with the steroid-naïve asthmatics or controls [79]. Nonselective cyclooxygenase inhibitors elevate B4 and decrease prostaglandin E_2_ levels in EBC in COPD patients, whereas selective cyclooxygenase inhibitors have no effect [80].

 In nonsmoking COPD patients, there is elevation of exhaled CO levels and also during exacerbations but decreases during recovery, respectively, [44, 81]. Elevation of exhaled ethane levels in smoking COPD patients [82] and elevation of exhaled isoprene [83] levels which are correlated with cholesterol biosynthesis [8] and pentane [84] levels in normal smokers [85] were shown. Increased levels of exhaled pentane and ethane which are found in inflammatory diseases [8] and elevation of exhaled and nasal NO, but not pentane, were also demonstrated in the patients with obstructive sleep apnoea with active inflammation [86] including lower EBC pH [87]. Elevation of exhaled 8-isoprostane levels, a marker of oxidative stress [88] by doubled and about 3-fold in patients with mild and severe asthma, respectively, which correlated with the disease severity, irrespective of corticosteroid treatment, excepted reduction of malondialdehyde (MDA) levels with treatment [89]. Elevation of both EBC and plasma MDA levels is found in asthmatic patients [90]. A study revealed that EBC MDA was related to both changes in pulmonary function and air pollution exposure [91]. Reduced EBC chlorine and serum eosinophilic cationic protein concentrations in asthma patients with airborne pollen exposure were found in a study [92]. Significant rapid decreases in S-nitrosothiols levels and nitrite/nitrate ratios but no changes in EBC 8-isoprostane levels were observed in patients with mild asthma who were treated with budesonide [93]. Elevation of exhaled sulphur-containing compounds levels was observed in allograft rejection and hepatic failure [8]. Elevation of EBC 8-isoprostane levels was also observed in normal cigarette smokers and COPD patients with much greater extent [94], further elevated during exacerbations [76] and is associated with disease severity [93–97], and 8-isoprostane [98] and nitrite [99] levels may reflect the pulmonary emphysema extension. In smoking persons, measurement of biomolecules in sputum supernatants and EBC, cellular analysis of induced sputum, measurement of FE_NO_, and measurement of breath VOCs with electronic nose application which can differentiate healthy smoking individuals from healthy nonsmoking subjects [67, 100] are noninvasive biomarkers of pulmonary inflammation and oxidative stress in subjects with cigarette smoking [100]. EBC IL-6 [46], MDA [101], S-nitrosothiols [102], and nitrite [102] are elevated in patients with COPD, and there is further elevation of IL-6 [103], IL-1-beta [101], TNF-alpha [81], and H_2_O_2_ [104] during exacerbations compared with normal smokers. No significant differences for EBC erythropoietin (EPO) concentrations or correlation between EPO and TNF-alpha levels were observed in COPD patients except that TNF-alpha concentrations were significantly higher in COPD patients than in non-COPD patients [105]. A study on EBC metallic elements in COPD patients revealed higher levels of aluminum, cadmium, and lead and lower levels of copper and iron with particular interest of EBC copper levels because of their positive correlation with pulmonary function parameters [106]. 

 In stable and exacerbation CF patients, there are markedly elevation of exhaled CO levels and decrease after treatment. This indicates that exhaled CO level is a marker of CF severity [107]. In 2000, a study reported increased concentrations of EBC 8-isoprostane in stable CF patients compared with control subjects [108]. CF patients demonstrate increasing of exhaled ethane levels, which is significantly correlated with airway obstruction and increased exhaled CO levels [109]. CF patients with exacerbations or stable period showed elevated levels of nitrite, nitrate [110, 111], and nitrotyrosine (NT) [112] in EBC and sputum [113]. Elevation of EBC nitrite and S-nitrosothiols is shown in adult patients with more severe CF [114] compared to CF children [115]. A study revealed failing of EBC free 3-NT as a marker for oxidative stress in stable CF and asthma children [116]. A study on 12 exhaled VOCs in young CF patients showed a significantly higher levels of pentane and 2-propanol, lower levels of dimethyl sulphide, correlation with median forced expiratory volume in one second (FEV_1_) for toluene in these patients, and no discrimination of CF patients from healthy subjects for ethane levels [117]. These VOCs were not correlated with CF genotype, treatment with ICS and deoxyribonuclease, or atopic status [117]. EBC TNF-alpha, interferon-gamma, IL-2, IL-4, IL-5, and IL-10 levels can be detected in CF or asthma children [118] with reduction of EBC IL-8 levels and increasing of pH values after antibiotic treatment [119]. EBC pH values show a significant mean difference between asthmatics and nonasthmatics [120] and its variability is not influenced by clinical status changes [121]. Elevated concentrations of EBC sodium and chloride ions were shown in CF patients and correlated with disease severity and the sweat test (Balint et al., unpublished observation). The percentages of methane producers in CF patients are higher than in normal subjects, but there is no difference between methane producers and nonproducers with respect to the degree of malabsorption among CF patients [122]. Glucose-hydrogen breath test after an overnight fast more likely shows elevation of fasting exhaled hydrogen levels compared with controls suggesting a high prevalence of small bowel bacterial overgrowth in these patients [123]. Use of inhaled ipratropium and laxatives is associated with a decreased risk of positive glucose-hydrogen breath test, whereas the use of azithromycin shows an increased risk [123]. 

 Increased concentrations of NO, CO, ammonia, pentane [51], ethane [51], nitrite, H_2_O_2_, 3-NT, adenosine [124], and 8-isoprostane are found in EBC in inflammatory pulmonary diseases including pneumonia, bronchiectasis, lung transplantation, idiopathic pulmonary fibrosis, and adult respiratory distress syndrome (ARDS) [44–127]. Elevation of exhaled ethane levels is correlated with disease severity and activity in interstitial lung disease patients [128]. A study on bronchiolitis obliterans syndrome (BOS) developed after lung transplantation revealed that helium slope had better sensitivity for detection of stages 0-p and 1 BOS than either exhaled NO or CO levels and the best sensitivity was observed with the three combined markers [129]. A recent study in lung transplant recipients indicated that the EBC pH variability was relatively small and similar to that in healthy nontransplant subjects [130]. Small amounts of exhaled ethane may be formed in the airways of asthmatic patients, whereas the major fractions of exhaled ethane, pentane, and isoprene seem to be of systemic origin [131]. Elevated EBC 3-NT [132] levels were also observed in patients with asthma including elevated EBC H_2_O_2_ [133] levels in patients with severe and unstable asthma. Whether bronchiectatic patients are treated with ICS or not, exhaled CO levels are elevated in them [134]. Exhaled NO levels seem to be decreased among patients with bronchiectasis infected with *P. aeruginosa* [135]. Increasing of exhaled CO and reduction of exhaled NO levels compared with patients without pulmonary hypertension and control subjects were observed in patients with systemic sclerosis [134–138]. However, the NOS3 levels in pulmonary vessels are variable [139–142]. Increasing of exhaled CO and NO levels was demonstrated in patients with fibrosing alveolitis [136, 143] and associated with disease activity [144]. The elevation of exhaled NO concentration originates from the alveolar level in the patients with alveolitis and from the bronchial level in the patients with asthma [60]. Either normal [145] or increasing [146] exhaled NO levels including elevation of EBC nitrite and nitrate concentrations [145] were reported in patients with active pulmonary sarcoidosis and reduced [146] by steroid treatment. A study in patients with newlydiagnosed pulmonary sarcoidosis showed that EBC TNF-alpha, plasminogen activator inhibitor 1, insulin-like growth factor 1, and BAL specimens were comparable and closely positively correlated [147]. In contrast, EBC IL-6 level was significantly lower compared with BAL level [147]. A recent study in sarcoidosis patients revealed that there were positive correlations between EBC and BAL LT B4 levels and 8-isoprostane levels, higher EBC and BAL cysteinyl leukotriene (cysLT) and 8-isoprostane levels compared with normal subjects, positive correlations between EBC LT B4 levels and the number of BAL lymphocytes per milliliter and between the number and percentage of BAL eosinophils and EBC 8-isoprostane and BAL cysLT levels, and no correlation between EBC eicosanoid levels and BAL macrophages, BAL lymphocytes, or superoxide production percentages [148]. A previous study revealed that liquid chromatography (LC) in combination with mass spectroscopy can accurately and quantitatively assess eicosanoids in EBC, particularly LT B4 and 8-isoprostane in patients with asthma and other respiratory diseases [149]. Some previous studies demonstrated that the combination of gas chromatography or LC and nuclear magnetic resonance (NMR) spectroscopy metabolomics profiling of EBC can increase sensitivity and specificity of the EBC analysis of small molecular weight metabolites [150, 151]. NMR spectroscopy metabolomics applied to EBC can characterize airway biochemical fingerprints, particularly in asthma pathophysiology [152]. 

## 6. Exhaled Markers in Critically Ill Patients

 A study among patients with head injury, ARDS, and those being at risk of developing ARDS concluded that plasma concentrations of MDA [153] and thiobarbituric acid-reactive substances [153] as well as breath concentrations of pentane [51, 153] and ethane increased with increasing inflammatory status. Exhaled hydrocarbon concentrations may be affected by contamination from bacterial flora in the gastrointestinal tract [51]. Acetone concentrations which are correlated with lipolysis [8] and dextrose metabolism [8] are not different between patient groups [85]. Significant correlation between exhaled breath acetone which is the most abundant human breath VOC [117] and propanol levels is seen in normal subjects [154] and elevation of exhaled acetone levels is observed in healthy subjects during exercise [155]. Isoprene concentrations are lowest in the ARDS group [153]. When compared with critically ill mechanically ventilated patients without pulmonary infection, there are reduced isoprene and increased pentane eliminations in these patients with pulmonary infection [156]. After cardiopulmonary bypass in adult patients, association of reduced exhaled NO levels with the decrease in pulmonary compliance and the increases in alveolar arterial pressure and pulmonary artery may indicate ARDS [157]. Significant elevation of EBC H_2_O_2_, hydrogen ions, and LT B4 levels was demonstrated in patients after pulmonary lobectomy [158]. Summary of studied volatile organic compounds is shown in Table 1.

## 7. Discussion

Several of the commonly occurring VOCs are derived from metabolic pathways that have been previously reported [163], for example, isoprene from the mevalonic acid pathway of cholesterol synthesis [163] and alkanes which are markers of oxygen free radical (ORF) activity in vivo [164]. ORF's degrade biological membranes by lipid peroxidation, converting polyunsaturated fatty acids to alkanes which are excreted through the lungs as VOCs [164]. A breath test for C4 to C20 alkanes and monomethylated alkanes provides a rational set of markers that identified pulmonary cancer in a group of patients with histologically proven disease [26]. It is limited by the following three main factors: the limited range of presenting disorders among the patients, the comparatively small number of patients with pulmonary cancer, and the comparative large number of variables in the breath-methylated alkane contour [26], a three-dimensional marker of oxidative stress [165]. 

The screening method must have acceptable technical performance parameters and must detect the disease at an earlier stage than would be possible without screening, while minimized false-positive and false-negative results [26]. In addition, early detection must improve disease outcome, cost, feasibility, and acceptability of screening and early treatment should be established [26]. The breath test is comparatively acceptable to patients, technically feasible, and low in cost [26]. 

Bronchial biopsies provide mediators and inflammatory cells, as well as the spatial relationships between the inflammatory processes in the airway wall, but they may not reflect all pathologic changes in the lung periphery, and the bronchoscopic invasiveness precludes repeated measurements. BAL may provide more information on small airway and alveolar inflammation, but it has variable dilution and invasiveness. Induced-sputum examination can provide information about mediators, cells, and markers of oxidative/nitrative stress, but it has high variability of the biomarkers. 

High reproducibility of FE_NO_ measurements within a single day in both children and adults is superior to any conventional methods of airway inflammation in asthma that may allow physicians to perform two instead of three exhalations for obtaining the reliable results [166] and reduce the cost of medical care [93]. This leads to their strong association with airway inflammation [159], asymptomatic asthmatic patients [167], insensitivity to beta-2 agonists [159], their high sensitivity to steroid treatment [168] which may significantly reduce the cost of research and medical care [93], noninvasiveness [166], and minimally staff-training procedures [166]. FE_NO_ does require an extra encouragement, compared with peak expiratory flow measurements [169]. Systemic errors or “learning effects” of serial FE_NO_ measurements are not found [170]. The measurements will not be accepted unless they are performed by following the guidelines [171]. A study showed requirement of only a small number of asthmatic patients for demonstration of a 25–80% effect of a studied drug in clinical trial [170]. When the FE_NO_ levels are below or above a certain reference level, steroid treatment should be either increased or reduced [172]. A study in lifelong never-smoking adults recently revealed that the geographic mean of FE_NO_ for the whole population was 16.6 parts per billion (ppb) and the upper limits of FE_NO_ ranged from 24.0 to 54.0 ppb [173]. Height and age would account for 9 to 11% of the variance of reference values [174]. Such upper FE_NO_ values are definitely higher than those reported in both normal adults and children in American Thoracic Society/European Respiratory Society (ATS/ERS) guidelines [175] and in a study by Olivieri et al. [176]. Recently, a study reported that FE_NO_ was significantly higher in patients with chronic rhinosinusitis and allergic rhinitis compared to patients with nonallergic rhinitis [177]. Seasonal variations of FE_NO_ values due to fluctuations of exposure to allergens have been reported [174]. According to this hypothesis, the measurements should have been reported whether performed outside or during pollen season [178]. With asthma and rhinitis having a same weight in FE_NO_ changes as recently reported in a random community survey of adults [179]. Probably missing in statistical analysis [173] or in replies to the questionnaire [180] of the high prevalence of nasal symptoms and/or allergic rhinitis and dietary consumption of fats in asthmatic children assuming low levels of antioxidants [181]. These could be important confounders for FE_NO_ reference values. NO chemiluminescence analyzers are mainly available in academic research laboratories and are presently expensive [166]. However, due to advances in technology, eventually it may be possible to introduce these devices in patients' homes [166]. Recently, FE_NO_ measurement with a portable analyzer has been used beneficially for young adults with asthma screening [182] including handheld device for FE_NO_ measurements in asthmatic children [178]. Therefore, repeated FE_NO_ measurements will not interfere with the system and be able to be used much more frequently, in contrast to the currently used semi-invasive or invasive procedures [159]. It is likely that the methods for nasal NO measurement need to be substantially standardized because of the high nasal NO levels from constitutive sources in the nose [183]. Flushing the nose with helium may reduce contamination of exhaled breath with nasal air that contains high NO levels, which may potentially influence the results of NO-related markers (S-nitrosothiols, nitrite/nitrate) [110]. One of the serious limitations of the exhaled aliphatic hydrocarbon [184] measurement is its requirement of large sample volume [185]. Today, there are a small number of researches using the hydrocarbon breath test as a marker of human lipid peroxidation [159, 164], but a study showed nonspecific [32]. The main reasons for the limited use of this method are technical difficulties [166]. The scrupulous avoidance of air contamination, the use of the right materials, an appropriate washout period, adequate preinjection concentrations of the samples, and a sensitive gas chromatographic technique enable the reproducible and accurate human breath hydrocarbon measurement [166].

 There is strong evidence that EBC composition abnormalities may reflect biochemical changes of airway lining fluid and potentially be used to measure the targets of modern therapy in COPD and asthma clinical trials [6]. Recently, most of the centers use a commercially available condenser that allows to collect a small volume of EBC within a few minutes and is equipped with a spirometer to register the exhaled air volume [75, 186, 187]. However, there are reports that cysLTs were undetectable in the EBC collected with a commercially available condenser [188]. It is unclear if this was due to the technical characteristic of this condenser, but another commercially available condenser can easily detect EBC cysLT in patients with asthma, both adults [73] and children [57], and COPD [69]. A recent Japanese asthma study showed significant correlation between urine LT E4 and EBC cysLTs levels in low-dose ICS treated patients, significant correlation between FE_NO_ and EBC cysLTs in steroid-naïve asthmatics, and no correlation between EBC cysLTs and FEV_1_, or log PC20Arc [161]. EBC LT B4 levels in patients with various pulmonary diseases [189] and NT levels in asthmatic [72] and CF [190] patients were validated by reversed-phase high-performance liquid chromatography. There is no significant difference between EBC H_2_O_2_ from neonates from a background with nasal continuous positive airway pressure alone or ventilator [191]. The spectrophotometric method commonly used to measure EBC H_2_O_2_ levels lacks specificity in ARDS due to the presence of variable levels of sample contaminants [192]. An elevation in calcium and a deficiency in magnesium concentrations in EBC have been reported in atopic asthma [162] as well as elevated EBC lactic acid levels in patients with acute bronchitis [193]. Identification and measurement of EBC proteins are controversial [194]. TNF-alpha, IL-1-beta, soluble IL-2 receptor protein, and IL-6 in EBC of patients with various respiratory conditions have been reported [162]. Higher concentrations of total EBC protein have been shown in young smokers compared with nonsmokers, whereas TNF-alpha and IL-1-beta levels were not different [160]. EBC IL-8 levels are more than doubled in unstable CF patients but are mildly increased in stable CF (Balint et al., unpublished observations). EBC collection can be successfully applied in healthy and asthmatic children with 100% success rate and negligible fall in FEV_1_ [195]. Although ratios of EBC compounds can provide insights into pulmonary redox status, issues of dilution remain critically important for the understanding of exact chemical constituents of the airways [196]. EBC can supplement FE_NO_ to more fully elucidate the nitrogen oxide chemistry of the lungs and airways [196]. EBC assays may monitor inflammation, oxidative stress, and acid stress [196]. A study revealed that EBC hydrogen ions, H_2_O_2_, and LT B4 elevated significantly in patients after pulmonary lobectomy, but not after the milder insult associated with cardiac surgery [158]. This indicates that EBC is a safe, noninvasive method of sampling the milieu of the distal lung and is sufficiently sensitive to detect markers of oxidative stress and inflammation in adults [166]. ATS/ERS Task Force on EBC provides the following general recommendations for 1-2 milliliters of oral specimen collection: collect during tidal breathing using a saliva trap and nose clip; 10 minutes collection time and define cooling temperature; use condenser with inert material; do not use filter and resistor between the condenser and subject [197]. A study suggested that tidal and minute volumes can predict the expected amount of collected EBC both in normal and airway disease subjects [198]. EBC represents a larger respiratory tract portions than BAL [197]. Comparison of EBC and BAL biomarkers in patients undergoing bronchoscopy for clinical indications in a study showed significantly higher pH, nitrogen oxides, and 8-isoprostane in EBC than in BAL, no different levels of H_2_O_2_ in EBC and BAL, significantly higher BAL protein levels, higher EBC phospholipid levels, and no different levels of keratin in EBC and BAL [199]. These indicate that the exact origin of EBC substances from different parts of the respiratory tract to each biomarker is still unknown [199]. Choice of dilutional marker is debatable [199]. There is yet no consensus on whether urea, electrolytes, or conductivity may be useful [199]. EBC has no introduced external factors compared with BAL [199]. Thus, EBC dilutional factors should be easier to assess [199]. Both techniques have significant potential limitations in addition to a significant reduction of exhaled NO levels with unknown underlying mechanisms led by bronchoscopy [200] and EBC sampling cannot be directly compared with BAL information [199]. Solubility, volatility, electric charges of EBC biomarkers, and collection technique may be involved [199]. The important areas of rapidly evolving EBC information for future studies involve ascertaining site and mechanisms of EBC particle formation, improving reproducibility, determination of dilution markers, EBC employment in longitudinal studies, determining the utility of EBC measures for the management of individual patients [197], and the cellular source of airway inflammation [201]. 

A study demonstrated a fundamental flaw in the breath pentane assays: the column employed in the gas chromatography did not separate pentane from isoprene, the most abundant compound in the breath [202]. What Mendis and colleagues reported in 1995 [203] as breath pentane was probably a mixture of pentane and isoprene [202]. A study noted that the details of Sobotka PA and colleagues' analytical technique that reported in 1994 [202] were sketchy; they may not have really been observing isoprene because most chromatographic columns do not separate pentane from isoprene [202, 204]. Increased clearance of alkanes and methylalkanes by CYP enzymes-induced smoking is consistent with previous reports that tobacco smoking is a potent inducer of CYP enzymes [205–207]. Increased circulating levels of conjugated dienes [208] and MDA [207, 209] in patients with pulmonary TB which indicated both local and systemic oxidative stress [210] were reported in addition to increased many breath VOCs among these patients [9] while existed only local reaction by slightly elevated levels of EBC hydrogen peroxide and thiobarbituric acid-reactive substances in sarcoidosis patients [210]. Currently, there is yet no single exhaled marker sufficiently sensitive and specific for diagnosis of sarcoidosis [211]. It may be assumed that serum total reactive oxygen metabolites levels can be used as an activity criterion in the differentiation of active and sequel pulmonary TB [212]. Sensitivity and specificity of pulmonary TB diagnosis can be increased with GC-MS breath tests [15, 16]. 

Currently, there is relative lack of information about how theses biomarkers are related to their reproducibility, disease severity, outcomes of concurrent therapies, and other clinical outcomes. None of the exhaled markers are diagnostic for a particular pulmonary disease, apart from very low exhaled and nasal NO in PCD. Single exhaled markers are usually evaluated in isolation, but markers are affected differently in different diseases, and their sensitivity varies to certain techniques. For example, as indicated above, COPD is characterized by normal or little elevation in exhaled NO, and by greater elevation in exhaled 8-isoprostane and CO levels [159]. By contrast, CF patients typically show low exhaled NO and high exhaled 8-isoprostane and CO levels [159], whereas asthma is characterized by a large elevation in exhaled NO, a moderate elevation in exhaled 8-isoprostane and CO [159], and elevation in EBC LT E4 levels which is not detectable in COPD [71]. Exhaled NO levels appear to be more sensitive to inhibition by low-dose ICS in asthma than exhaled 8-isoprostane and CO levels [159]. Nevertheless, monitoring of these markers may help differential diagnosis of pulmonary diseases. The choice of which biomarker is measured will depend on the clinical problem that is being addressed or posted; research questions for example, prediction of steroid responsiveness may be provided by increased sputum eosinophils and FE_NO_. The use of FE_NO_ measurements may minimize the potential long-term side effects of ICS [213, 214]. FE_NO_ analyzers are approved by the United States Food and Drug Administration for assessing and monitoring therapy in asthmatic patients, particularly with eosinophilia and patients with airway inflammation [214]. FE_NO_ measurement can reduce approximately 40% of daily dose of inhaled glucocorticoids with similar efficacy and reduce costs [214]. Nevertheless, FE_NO_ is only one surrogate biomarker of airway inflammation, but unlikely to ruminate the whole complexity of the inflammatory process in respiratory diseases and its multiple expressions within a given syndrome or disease [214]. Omics technologies (genomics, transcriptomics, proteomics, lipidomics, breathomics, and metabolomics), a novel omics technology that is purposed at detecting and quantifying breath biomolecules by reference analytical techniques and detecting selective profiles of breath VOCs by electronic nose and that requires close interdisciplinary interactions (biophysics, bioinformatics, and bioengineering), could have important implications for the pharmacological therapy of respiratory diseases [214]. With identification of subphenotypes of patients with respiratory diseases, it would facilitate the implementation of a personalized and tailored pharmacotherapy [214]. The omics technologies could also have therapeutic implications in asymptomatically maintained lung-function patients with ongoing pulmonary inflammation who might require starting pharmacological treatment [214]. Conclusions of randomized clinical trials are whether omics technologies able to apply to large patient populations, but they are still the best tool for evaluation of the pharmacological profiles of novel and existing drugs [214]. Most researches have now focused on markers in asthma; some data refer to infections, CF, and PCD surrogate markers [215]. The use of FE_NO_ measurement in treatment decision costs less than asthma management based on standard guidelines and has similar health benefits [216]. Although an exhaled NO elevation is not specific for asthma, the exhaled NO measurement can be used in distinguishing asthma from other obstructive airway disease conditions [217]. Elevated exhaled NO may indicate reflects some, but not all, aspects of airway inflammation, and needs further studies to determine how it relates to some other airway inflammatory markers (e.g., IL-4, IL-5, IL-6, IL-8, IL-10, and TNF-alpha) [159]. *Cryptococcus neoformans* is a pathogenic fungus that produces large amounts of urease which catalyzes the hydrolysis of urea to ammonia that can be detected in exhaled breath [218]. It is possible that exhaled ammonia measurements might differentiate bacterial and viral infections in a variety of pulmonary diseases [159]. 

In the past, H_2_O_2_ was the most studied substance on numerous inflammatory diseases as a marker of oxidative stress [219]. Today, exhaled NO has been studied the most and its relevance to clinical practice markers of inflammation into daily practice should be evaluated [209]. There is a pressing need for the evaluation of these techniques in long-term clinical studies [5]. In the future, each disease may have a fingerprint or profile of different markers that may be diagnostic. Possibilities for measurement of exhaled breath markers are far greater than presently realized. Application of proteomics with high resolution two-dimensional gel electrophoresis and microanalysis of protein spots may allow the recognition of particular protein patterns in various diseases including malignancies and the origin of EBC proteins [199] and may result in the recognition of new therapeutic targets, many other markers of inflammation, and even specific fingerprints of activation or diagnostic proteins [159]. 

Measurement of exhaled breath temperature and humidity is another method which may serve as a simple, non-specific, and inexpensive method for home monitoring of rhinitis, COPD, CF, and asthma including assessment of anti-inflammatory treatment effects [159]. A study supported the hypotheses that exhaled breath temperature is associated with the degree of airway inflammation in asthma [220]. EBC temperature influences condensate pH but not total protein content [221]. Condenser type influences total protein content, cysLT concentration, and sample pH [221]. These indicate that EBC temperature and adherence of the biomarkers to condenser surface may play a role but does not fully explain the variability of EBC biomarker levels [221]. So EBC collection temperature should be controlled and reported [222]. EBC standardized guidelines should include the most valid condenser coating for specific biomarker measurement [223]. For example, a condenser with glass or silicone coating is more efficient for albumin or 8-isoprostane measurement [223]. Liquid chromatography-electrospray ionization tandem mass spectrometry (LC/MS/MS) which has been used to analyze exhaled LT B4 levels in asthmatic children serves as a fast and easy method to assess the utility of EBC biomarkers for differentiation of pulmonary diseases [224] and is potentially suitable for longitudinal studies and drug-therapy assessment in patients with pulmonary diseases, but a presently important limitation is its high cost [201]. Recently, LC/MS/MS method has been used successfully to detect EBC urea, adenosine, adenyl purines, and adenosine monophosphate (AMP) and elevated AMP/urea ratios in CF patients [225]. Laser magnetic resonance spectroscopy can be a simple alternative to mass spectrometry in detection of exhaled ^14^C-urea in *Helicobacter pylori* infection [226, 227]. A method of gas chromatography combined with ultraviolet spectroscopy has been used to measure exhaled acetone and isoprene levels in diabetic school children [228] and to monitor pulmonary cancer-cytostatic chemotherapy efficacy [39]. Polymer-coated surface-acoustic-wave resonators are portable instruments which have been introduced and are able to analyze organic vapors [229]. The selected ion flow tube technique for trace gas analysis of air and breath is sensitive and can be used during a normal breathing cycle [230]. 

## 8. Conclusions

 Measurements of exhaled hydrocarbon and other exhaled markers are much more difficult using current technology. EBC value will depend on availability of reliable, fast, and inexpensive detector systems and standardized collection that will overcome its high cost and its present highly variable measurements. This could have far-reaching potential for the diagnosis and treatment of various respiratory diseases. There is little doubt that “inflammometry” will be a major step forward and will be useful in differentiating airway diseases and improving treatment. In the future, it is likely that smaller and more sensitive analyzers will extend the discriminatory value of exhaled breath analysis and that these techniques may be available to diagnose and monitor respiratory diseases in the general practice and home setting. More detailed insights into inflammatory processes can be obtained when the volatile and serum markers are considered together. The evaluation of FE_NO_ reference values in the general population must consider dietary habits, age, weight, height, gender, asthma, atopy, smoking habits, current nasal and respiratory symptoms, and steroid assumption. The effect of various factors and different subpopulations on healthy subjects is also reported, in an effort to delineate future directions that need the establishment of EBC reference values. 

## Figures and Tables

**Table 1:** Summary of studied diseases, studied volatile organic compounds and references.

Studied diseases	Studied exhaled breath* and in vitro volatile organic compounds and exhaled breath condensates**		References
	Breath	Culture (in vitro)	
Pulmonary tuberculosis	(1) Benzene, ethyl-* (2) Benzene, methyl-* (3) Benzene, propyl-* (4) Heptane, 3-methyl-* (5) Propane, 2-methoxy-2-me* (6) 1-Octene* (7) Cyclohexane* (8) Heptanal* (9) Heptane, 2,4-dimethyl-* (10) Heptane, 4-methyl-* (11) Nonanal* (12) Pentane, 2-methyl-* (13) Styrene* (14) Tridecane* (15) Cyclohexane, 1,3- -dimethyl-, trans-* (16) Benzene, 1,4-dichloro-* (17) Cyclohexane, 1,4- -dimethyl-* (18) 1-Octanal, 2-butyl-* (19) 2-Butanone* (20) Naphthalene, 1-methyl-* (21) Camphene* (22) Decane, 4-methyl-* (23) Heptane, 3-ethyl-2- methyl-* (24) Octane, 2,6-dimethyl-* (25) Benzene, 1,2,3,4- -tetramethyl* (26) Icycle_3_1_1_hept-2- -ene, 3,6,6-trimethyl-* (27) Cyclohexane, 1-ethyl-4-methyl-, trans-* (28) 1-_beta_-Pinene*	(1) Naphthalene, 1-methyl- (2) 3-Heptanone (3) Methylcyclododecane (4) Heptane, 2,2,4,6,6- pentamethyl- (5) Benzene, 1-methyl-4- -(1-methylethyl)- (6) Cyclohexane, 1,4-dimethyl- (7) 3,5-Dimethylamphetamine (8) Butanal, 3-methyl- (9) 2-Hexene, trans-anti-1-methyl- -decahydronaphthalene	[9, 15–18, 24]
	(1) Hydrogen peroxide** (2) Nitric oxide* (3) Carbon dioxide* (4) Ammonia*		[10–13, 22, 23, 25]

Pulmonary cancer	(1) 1,5,9-Cyclododecatriene, 1,5,9-trimethyl-* (2) Pentan-1,3-dioldiisobutyrate, 2,2,4-trimethyl-* (3) Benzoic acid, 4-ethoxy-, ethyl ester* (4) Propanoic acid, 2-methyl-, 1-(1,1-dimethylethyl)-2- -methyl-1,3-propanediyl ester* (5) 10,11-Dihydro-5H-dibenz-(B,F)-azepine* (6) 2,5-Cyclohexadiene-1,4-dione, 2,6-bis(1,1-dimethylethyl)-* (7) Benzene, 1,1-oxybis-* (8) Furan, 2,5-dimethyl-* (9) 1,1-Biphenyl, 2,2-diethyl-* (10) 3-Pentanone, 2,4-dimethyl-* (11) trans-Caryophyllene* (12) 1H-Indene, 2,3-dihydro-1,1,3-trimethyl-3-phenyl-* (13) 1-Propanol* (14) Decane, 4-methyl-* (15) 1,2-Benzenedicarboxylic acid, diethyl ester* (16) 2,4-Hexadiene, 2,5-dimethyl-* (17) Isoprene (1,3-butadiene, 2-methyl-)* (18) 2-Methylpentane* (19) Pentane* (20) Ethylbenzene* (21) Xylenes* (22) Trimethylbenzene* (23) Toluene* (24) Pentamethylheptane* (25) Ethynylbenzene* (26) Heptane, 2,2,4,6,6-pentamethyl-* (27) Heptane, 2-methyl-* (28) Benzene, propyl-* (29) Undecane* (30) Cyclopentane, methyl-* (31) Cyclopentane, 1-methyl-2-pentyl-* (32) Methane, trichlorofluoro-* (33) Benzene, 1,2,4-trimethyl-* (34) Octane, 3-methyl-* (35) 1-Hexene* (36) Nonane, 3-methyl-* (37) 1-Heptene* (38) Benzene, 1,4-dimethyl-* (39) Heptane, 2,4-dimethyl-* (40) Hexanal* (41) Cyclohexane* (42) Benzene, 1-methylethenyl-* (43) Hepatanal* (44) Styrene* (45) Propyl benzene* (46) 1,2,4-Trimethyl benzene* (47) Heptanal* (48) Methyl cyclopropane* (49) Acetone* (50) Tumor necrosis factor-alpha** (51) Interleukin-2** (52) Leptin** (53) Deoxyribonucleic acid** (54) Matrix metalloproteinase-9**		[26–34, 36–39]
	Nitric oxide*		[27, 35]

Respiratory infections/infestations			
*Pseudomonas aeruginosa *	(1) Hydrogen cyanide* (2) Nitric oxide*		[43, 135]
*Staphylococcus aureus *	Nitric oxide*		[48]
Bacterial	(1) Nitric oxide* (2) Carbon monoxide* (3) Volatile fatty acids (BAL)		[44, 48–50, 54]
Viral	(1) Nitric oxide* (2) Carbon monoxide*		[45–48]
Parasitic	Nitric oxide*		[48]
*Streptococcal pneumoniae* *Aspergillus niger* *Aspergillus terreus* *Aspergillus flavus* *Aspergillus fumigatus* *Fusarium *species	2-Pentylfuran*		[53]

Primary ciliary dyskinesia	Nitric oxide*		[57]

Cystic fibrosis	(1) Nitric oxide* (2) Carbon monoxide* (3) Hydrogen* (4) Pentane* (5) Ethane* (6) Methane* (7) Dimethyl sulphide* (8) Toluene* (9) 2-Propanol* (10) 8-Isoprostane^∗, ∗∗^ (11) Nitrite** (12) Nitrate** (13) Nitrotyrosine** (14) S-Nitrosothiols** (15) Tumor necrosis factor-alpha** (16) Interferon-gamma** (17) Interleukin-2** (18) Interleukin-4** (19) Interleukin-5** (20) Interleukin-8** (21) Interleukin-10**		[48, 57, 63, 107–123, 159, 160]

Asthma	(1) Nitric oxide* (2) Nitrite** (3) Nitrate** (4) 3-Nitrotyrosine** (5) S-Nitrosothiols** (6) Malondialdehyde** (7) Carbon monoxide* (8) Hydrogen peroxide** (9) Ammonium ions** (10) Leukotrienes B4**, C4**, D4**, E4**, F4** (11) Cysteinyl leukotrienes** (12) Thromboxane B2-L1** (13) Nepsilon-(carboxymethyl)lysine** (14) Eotaxin** (15) Macrophage-derived chemokine** (16) Pentane* (17) Ethane* (18) Isoprene* (19) 8-Isoprostane^∗, ∗∗^ (20) Calcium ions** (21) Magnesium ions** (22) Chlorine**		[57, 60–75, 78, 79, 89, 92, 93, 131–133, 159, 161, 162]

Chronic obstructive pulmonary disease	(1) Nitric oxide* (2) Nitrite** (3) S-Nitrosothiols** (4) Hydrogen peroxide** (5) Carbon monoxide* (6) Prostaglandin E_2_*, F_2_-alpha** (7) Interleukin-1-beta** (8) Interleukin-6** (9) Tumor necrosis factor-alpha** (10) Leukotriene B4** (11) Cysteinyl leukotrienes** (12) Malondialdehyde** (13) Erythropoietin** (14) Ethane* (15) Isoprene* (16) Pentane* (17) 8-Isoprostane^∗,∗∗^ (18) Aluminum** (19) Cadmium** (20) Lead** (21) Iron** (22) Copper**		[8, 44, 57–60, 69–71, 74, 76, 80–84, 93–106, 159]

Pneumonia Bronchiectasis Lung transplant rejection Idiopathic pulmonary fibrosis Adult respiratory distress syndrome Inflammatory pulmonary diseases	(1) Nitric oxide* (2) Nitrite** (3) 3-Nitrotyrosine** (4) Carbon monoxide* (5) 8-Isoprostane** (6) Pentane* (7) Ethane* (8) Hydrogen peroxide** (9) Ammonia* (10) Helium* (11) Adenosine**		[44–127, 129, 158]

Obstructive sleep apnoea with active inflammation	(1) Nitric oxide* (2) Pentane* (3) Ethane*		[86]

Systemic sclerosis Fibrosing alveolitis Pulmonary sarcoidosis	(1) Nitric oxide* (2) Carbon monoxide* (3) Ethane* (4) Nitrite** (5) Nitrate** (6) Insulin-like growth factor 1** (7) Plasminogen activator inhibitor 1** (8) Tumor necrosis factor-alpha** (9) Interleukin-6** (10) Cysteinyl leukotrienes** (11) Leukotriene B4** (12) 8-Isop[rostane**		[128, 134–148]

Asbestosis	(1) Nitric oxide* (2) 8-Isoprostane** (3) Leukotriene B4**		[55, 56]

Silicosis	(1) 8-Isoprostane** (2) Leukotriene D4**		[58]

Adult respiratory distress syndrome Head injury	(1) Nitric oxide* (2) Pentane* (3) Ethane* (4) Isoprene* (5) Acetone* (6) Hydrogen peroxide**		[51, 124, 127, 153, 155–157]

Pulmonary lobectomy	(1) Hydrogen peroxide** (2) Hydrogen ions** (3) Leukotriene B4**		[158]
